# The brain is defenseless against pesticides

**DOI:** 10.1590/1806-9282.712EDIT

**Published:** 2026-05-01

**Authors:** Fulvio Alexandre Scorza, Antonio-Carlos Guimarães de Almeida, Josef Finsterer, Marcelo Feijó de Mello

**Affiliations:** 1 Instituto Israelita de Ensino e Pesquisa, Hospital Israelita Albert Einstein, Albert Einstein Israelite Faculty of Health Sciences – São Paulo (SP), Brazil.; 2 Universidade Federal de São Paulo, Escola Paulista de Medicina, Department of Neurology and Neurosurgery, Discipline of Neuroscience – São Paulo (SP), Brazil.; 3 Universidade Federal de São João del-Rei, Bioengineering Program, Laboratory of Experimental and Computational Neuroscience – São João del-Rei (MG), Brazil.; 4 Neurology and Neurophysiology Center, Department of Neurology – Vienna, Austria.; 5 Universidade Federal de São Paulo, Escola Paulista de Medicina, Department of Psychiatry – São Paulo (SP), Brazil.

Nutrition is an integral part of solving many of the world’s medical, societal, environmental, and economic challenges^1^. Furthermore, nutrition is essential for vitality and is a critical part of health and development^1^. Based on these data, we would like to add some thoughts that may open the debate on the use of pesticide residues in food and their direct or indirect effects on brain health.

In the last few decades, agriculture has become increasingly dependent on the synthetic chemical industry, which produces patented seeds, fertilizers, and pesticides^2^. Under these conditions, humans and their environment have been strongly contaminated by synthetic chemical substances used in agriculture^2^. Lamentably, Brazil sets a record for pesticide consumption, accounting for approximately 20% of total global use^3^. Furthermore, our country is considered one of the largest importers of pesticides, being allowed the use of fifteen pesticides that are banned in the European Union^3^.

Additionally, it has been verified that several adverse health effects are associated with exposure to chemical pesticides, including gastrointestinal, respiratory, reproductive, endocrine, obstetric, carcinogenic, and neuropsychiatric consequences^2^. According to the last adverse effect, it has been shown that some pesticide families can cause severe damage to the nervous system and have been suspected of playing a role in the development of brain disorders^2,4^. In fact, the use of pesticides in Brazil represents a silent health crisis, affecting not only workers and farm laborers but also consumers of contaminated food^5^. Unfortunately, certain pesticides (e.g., carbamates, organochlorine compounds, and organophosphates) can cause severe brain damage and pose significant risk factors for the development of neuropsychiatric disorders^2,6,7^. These pesticides (primarily herbicides and insecticides) indirectly produce harmful neurological effects and unbalance the cellular, biochemical, and molecular mechanisms that maintain brain activity^7,8^. In this sense, individuals exposed to multiple pesticides may therefore develop cognitive impairments (memory, attention, and visuospatial processing), motor disorders, and an increased risk of neurological diseases such as Parkinson’s, Alzheimer’s, and amyotrophic lateral sclerosis^2,6,7,8^. Of particular note is that pesticide exposure among farm laborers increases the likelihood of anxiety and depression, thus more severely impacting their mental health^4,9^. From a pathophysiological point of view, it is likely that changes in synaptic structure and function play a crucial role in the development of these neuropsychiatric conditions^4,9^. Unfortunately, these mental disorders are linked to a high suicide rate among farmers (twice the national average), which is one of the biggest concerns for some farming communities^9^. Furthermore, it should be emphasized that chronic stress in agricultural communities, caused by climate uncertainties, financial pressures, social isolation, precarious housing conditions, and work-related challenges, directly impacts the mental health of farmers^10^.

Overall, the devastating effects of pesticides on mental health require greater attention, dialog, and cooperation between scientists, society, and authorities. However, what are the healthiest foods? Really, while some fruits and vegetables have clear evidence supporting their recommendations, others require careful evaluation due to the presence and levels of pesticide residues^11^. In fact, residues of pesticides can be found in a great variety of everyday foods and beverages, including, for instance, cooked meals, water, wine, fruit juices, refreshments, and animal feeds^11^. Putting all this data together, where do we go from here? First, our research group is also convinced that higher fruit and vegetable consumption plays a significant role in improving mental health status^12^. However, we must also be aware that ingestion of pesticide residues from fruits and vegetables, depending on the quantity and ways in which a person is exposed, is a serious hazard and deserves intense debate in the scientific community. Second, alongside the need for further clinical trials, it is vital to consider the consumption of organic food, as it significantly reduces consumers’ dietary pesticide exposure and the acute and chronic risks associated with it^13^. Despite the existence of a misguided culture that organic food tends to be more expensive than conventionally produced food^14^, organic products are healthy and should be strongly recommended. Meanwhile, people should further limit their intake of pesticide residues by peeling or washing fruit and vegetables, which also reduces other foodborne hazards such as harmful pathogens. These considerations show how important it is to put the issue of “pesticides and mental health” on the public agenda, also in view of its overlap with economic interests, infrastructure, and social and environmental policies. Therefore, the most important requirement is to educate our population that habitual consumption of organic foods often contains more beneficial nutrients, less pesticides and improves mental and physical health. Finally, scientists should work in convergence with health professionals to provide preventive information and develop treatment plans for individuals exposed to pesticides and, whenever possible, advocate for public policies that protect the health of the population and the environment.
